# Survival extrapolation in the presence of cause specific hazards

**DOI:** 10.1002/sim.6375

**Published:** 2015-03-05

**Authors:** Tatiana Benaglia, Christopher H. Jackson, Linda D. Sharples

**Affiliations:** ^1^ Department of Statistics Universidade Estadual de Campinas Sao Paulo Brazil; ^2^ Medical Research Council Biostatistics Unit Cambridge U.K.; ^3^ Clinical Trials Research Unit University of Leeds Leeds U.K.

**Keywords:** survival analysis, survival extrapolation, poly‐weibull, polyhazard, cause specific hazards

## Abstract

Health economic evaluations require estimates of expected survival from patients receiving different interventions, often over a lifetime. However, data on the patients of interest are typically only available for a much shorter follow‐up time, from randomised trials or cohorts. Previous work showed how to use general population mortality to improve extrapolations of the short‐term data, assuming a constant additive or multiplicative effect on the hazards for all‐cause mortality for study patients relative to the general population. A more plausible assumption may be a constant effect on the hazard for the specific cause of death targeted by the treatments. To address this problem, we use independent parametric survival models for cause‐specific mortality among the general population. Because causes of death are unobserved for the patients of interest, a polyhazard model is used to express their all‐cause mortality as a sum of latent cause‐specific hazards. Assuming proportional cause‐specific hazards between the general and study populations then allows us to extrapolate mortality of the patients of interest to the long term. A Bayesian framework is used to jointly model all sources of data. By simulation, we show that ignoring cause‐specific hazards leads to biased estimates of mean survival when the proportion of deaths due to the cause of interest changes through time. The methods are applied to an evaluation of implantable cardioverter defibrillators for the prevention of sudden cardiac death among patients with cardiac arrhythmia. After accounting for cause‐specific mortality, substantial differences are seen in estimates of life years gained from implantable cardioverter defibrillators. © 2014 The Authors *Statistics in Medicine* Published by John Wiley & Sons Ltd.

## 1 Introduction

In health economic evaluations, we compare the expected cost and clinical effectiveness associated with different treatments or other health technologies. In the UK, the organisation responsible for these appraisals is the National Institute for Health and Clinical Excellence, which recommends whether a new technology should be given public funding in the presence of a limited budget. An important component of many health economic evaluations is the effect of the new treatment on the long‐term survival or some functional of it, such as mean survival or life expectancy for the groups of interest. Treatment effects are usually obtained from RCTs. However, for many chronic diseases, the choice of treatment has an impact on survival over much longer periods than those covered by the follow‐up of trials. Typically, estimates of expected lifetime survival are required to evaluate each treatment policy, but only a short‐term survival curve is available from the trial.

Assuming that short‐term individual‐level data are available, the simplest approach to estimating long‐term expected survival is to fit a parametric or semi‐parametric survival model to these data, then integrate the fitted survival curve over a lifetime 1. This approach is often sensitive to the choice of model 2. More importantly, extrapolations from even the best‐fitting models may be unrealistic if the observed period is short compared to the unobserved period. Then it is desirable to include relevant external long‐term data. Survival data for patients with the disease of interest may be available from disease registries or hospital‐based cohorts 1. These may provide information on longer‐term follow‐up or older ages 3, but the follow‐up will not always be sufficiently long. Population lifetime survival might be obtained from national agencies such as the UK Office for National Statistics; however, untestable assumptions about how the general population survival differs from the extrapolated survival of the patients of interest are usually necessary. For example, patients who have survived a certain length of time may sometimes be assumed to be ‘cured’ and have the same survival as the general population 4.

Our motivating example concerns implantable cardioverter defibrillators for the secondary prevention of cardiac arrhythmia. Connolly *et al*. 5 present a meta‐analysis of RCTs comparing implantable cardioverter defibrillators (ICDs) to anti‐arrhythmic drugs (AAD). The studies included had around 5 years of follow‐up. To inform policy in a UK context, we combine their published summaries with individual data from a cohort of UK ICD patients and age‐sex matched national population survival data [available at http://www.ons.gov.uk/ons/taxonomy/index.html?nscl=Interim+Life+Tables]. Demiris and Sharples 6 described models for estimating expected survival based on these specific datasets. A Bayesian model with shared parameters was fitted simultaneously to all data sources. Hazards were assumed to be proportional (or additive) between the study populations and the general population. More plausibly, however, the relationships between the hazards will be different for different causes of death. In polyhazard models 7, the overall hazard is decomposed as a sum of contributions, interpreted as particular causes of death, which are not published in the data. Demiris *et al*. 8 used a poly‐Weibull model to extrapolate mean survival after transplantation, using a single dataset with reasonably long follow‐up, where high initial surgery‐related hazards and increasing long‐term mortality results in a ‘bathtub’ or U‐shaped hazard curve.

In this paper, we combine short‐term individual‐level data on patients of interest with causes of death unknown, and long‐term general population data with causes of death known, to obtain extrapolations of the expected lifetime of the patients of interest. We assume that the hazard for death from the disease of interest is proportional, while the hazard for all other causes is identical, between the study patients and the general population. In Section 2, we present the two‐component polyhazard modelling framework used throughout this paper. Then in Section 3, we perform a simulation study to investigate the biases in expected survival resulting from assuming that the hazard for all‐cause mortality is proportional between the general population and the study patients (as in 6) when in reality the relationships among the hazards are different for different causes of death. Section 4 presents the proposed methodology applied to the data studied by Demiris and Sharples 6 on the comparison between implantable cardioverter defibrillators and AAD for cardiac arrhythmia. As well as being proportional between the general and study population, hazards are also assumed to be proportional between treatment groups within the study population, although this assumption is also untestable given the limited follow‐up of the RCT providing the treatment effect. Finally, we discuss the findings and limitations of these methods, in particular the risks of combining data from different sources and the importance of publishing causes of death.

## 2 Two component polyhazard model

We have two individual‐level survival datasets, one from the general population and one from patients with some disease of interest. Suppose there are two possible causes of death, where the disease group has an increased hazard of death from the cause of interest *k* = 1 and identical hazard for other causes *k* = 2. In this section, we show how to use the population data to extrapolate the survival of the disease group. For the moment, we assume the patients of interest all receive the same treatment. In Section 4.3, we extend this method to a comparison of survival between treatment groups.

### 2.1 Population data

Assume that for an individual in the general population (superscript p), the distributions of the times to death $T_{k}^{p}$ for causes *k* = 1,2 come from the same parametric family but with different parameters. For illustration, here, we use Weibull distributions with shape and rate parameters *α*_*k*_ and *λ*_*k*_, respectively:

$$T_{k}^{p}∼\text{Weibull}(\alpha_{k},\lambda_{k}).$$

Thus, the hazard is either constant, increasing or decreasing over time if *α*_*k*_=1, *α*_*k*_>1 or *α*_*k*_<1, respectively. For each individual *i* in the population data, suppose the cause of death is known, so the corresponding cause‐specific survival time $t_{\mathrm{ik}}^{p}$ is observed if the individual died from cause *k* (denoted $\delta_{\mathrm{ik}}^{p}=1$) or censored otherwise $\left(\delta_{\mathrm{ik}}^{p}=0\right)$. The data $y_{k}^{p}=\left\{\left(t_{\mathrm{ik}}^{p},\delta_{\mathrm{ik}}^{p}\right):i=1,\ldots ,n\right\}$ for *k* = 1,2 therefore contribute the following terms to the likelihood for parameters *α* = (*α*_1_,*α*_2_) and *λ* = (*λ*_1_,*λ*_2_).

(1) $$L_{k}^{p}\left(\alpha ,\lambda |y_{k}^{p}\right)=\overset{n}{\underset{i=1}{\prod }}S_{p}\left(t_{\mathrm{ik}}^{p}|\alpha_{k},\lambda_{k}\right)h_{p}{\left(t_{\mathrm{ik}}^{p}|\alpha_{k},\lambda_{k}\right)}^{\delta_{\mathrm{ik}}^{p}},$$

where $S_{p}(t|\alpha_{k},\lambda_{k})=\exp\left(-\lambda_{k}t^{\alpha_{k}}\right)$ and $h_{p}(t|\alpha_{k},\lambda_{k})=\lambda_{k}\alpha_{k}t^{\alpha_{k}-1}$ are the survivor and hazard functions of the cause‐specific Weibull distributions.

### 2.2 Study data

For patients in the study group, we assume that the other‐cause survival distribution is the same as that for the population, Weibull(*α*_2_,*λ*_2_). The hazard for the cause of interest, however, is proportionally increased to reflect their higher risk, with the increase described by the cause‐specific log hazard ratio *β* between the study and population groups. Thus, the survival distribution for the cause of interest is Weibull (*α*_1_,*e* ^*β*^ *λ*_1_).

The causes of death are *not observed* in the study data; therefore, we cannot fit independent Weibull models as in (1). Instead, we use the fact that if there are *K* competing causes of death with respective hazard functions *h*_1_(*t*),…,*h*_*k*_(*t*), then the actual death time follows a *polyhazard* model 7 with overall hazard $\underset{k}{\sum }h_{k}(t)$. In this case, *k* = 1 or 2, and the observed death times in the study data follow a *poly‐Weibull* distribution with hazard function:

(2) $$\begin{array}{cc} h_{s}(t|\alpha ,\lambda ,\beta ) & =e^{\beta }h_{p}^{1}(t|\alpha_{1},\lambda_{1})+h_{p}^{2}(t|\alpha_{2},\lambda_{2})\\ =e^{\beta }\lambda_{1}\alpha_{1}t^{\alpha_{1}-1}+\lambda_{2}\alpha_{2}t^{\alpha_{2}-1}. \end{array}$$

The contribution of the study data $y^{s}=\left\{\left(t_{j}^{s},\delta_{j}^{s}\right):j=1,\ldots ,m\right\}$ to the overall likelihood is given by

(3) $$L^{s}\left(\alpha ,\lambda ,\beta |y^{s}\right)=\overset{n}{\underset{j=1}{\prod }}S_{s}\left(t_{j}^{s}|\alpha ,\lambda ,\beta \right)h_{s}{\left(t_{j}^{s}|\alpha ,\lambda ,\beta \right)}^{\delta_{j}^{s}},$$

where the survivor function for the poly‐Weibull distribution is $S_{s}(t|\alpha ,\lambda ,\beta )=\exp\left(-e^{\beta }\lambda_{1}t^{\alpha_{1}}-\lambda_{2}t^{\alpha_{2}}\right)$.

### 2.3 Combining population and study data

The overall likelihood for the combined population and study data is then

(4) $$L\left(\alpha ,\lambda ,\beta |y_{1}^{p},y_{2}^{p},y^{s}\right)=L^{s}\left(\alpha ,\lambda ,\beta |y^{s}\right)\overset{2}{\underset{k=1}{\prod }}L_{k}^{p}\left(\alpha ,\lambda |y_{k}^{p}\right).$$

We estimate the parameters *α*,*λ*,*β* in a Bayesian framework. This allows any previously published information about differences between populations to be included as prior distributions on the corresponding hazard ratio (Section 4.3) and facilitates computation by MCMC in software such as WinBUGS 9. During the MCMC process, we use samples from the posterior distributions of the parameters to compute the posterior distribution of the mean survival, or life expectancy of the study patients, defined as the area under the survival curve:

$$E(T^{s})=\overset{\infty }{\underset{0}{\int }}S_{s}(t|\alpha ,\lambda ,\beta )\mathrm{dt}.$$

Polyhazard models based on various distributions, including the Weibull, were compared by Louzada‐Neto 7. In applications, the fit of any assumed distribution to the observed data, and its plausibility in extrapolation, should be checked. An advantage of the Weibull (also shared by the Gompertz distribution, for example) is its proportional hazards property, which enables differences between populations to be expressed intuitively as hazard ratios. The potential identifiability problems with the poly‐Weibull model have been discussed 8—when fitted to a single survival dataset without cause of death recorded, the parameters can only be identified if the multiple causes have hazard trajectories that can be easily distinguished given the overall hazard trajectory. For example, two causes with strictly increasing and strictly decreasing hazards result in a U‐shaped hazard for overall survival. In our case, information on the cause‐specific parameters *λ*_*k*_ and *α*_*k*_ is enhanced by the population data $y_{k}^{p}$ through the likelihood (4). In Section 4.3, we explain how to extend this method to also estimate the survival of patients receiving a different treatment from those in the study data, assuming that a summary treatment effect is available from literature or further individual data.

## 3 Simulation

In this section, we perform a simulation study to investigate the potential bias incurred from ignoring a cause‐specific survival effect when extrapolating survival. Estimates obtained by the polyhazard‐based model described in Section 2 are compared with those from two misspecified models, which do not take into account the cause‐specific effect but assume that the study patients have a proportionally increased hazard for *overall* survival compared to the general population.

### 3.1 Design of the simulation study

Censored survival data for hypothetical general and study populations, with assumed age of 60 at time 0, were simulated. We assumed two mutually exclusive causes of death, each with a Weibull survival distribution, as in Section 2. This implies two‐component poly‐Weibull distributions for overall survival. The following factors that could affect the accuracy and precision of the parameter estimates were varied in the simulations:

1. The Weibull shape parameters *α* (Table 1). Model 1 assumes that the shape parameters for the two causes of death are the same, equivalent to a Weibull model for overall mortality with hazard equal to the sum of the component‐specific hazards. Models 2 and 3 have a higher shape parameter for causes other than the disease of interest, suggesting that the proportion of overall mortality due to the cause of interest decreases through time, and this is particularly rapid in Model 3.
2. The increase in the cause specific risk for the study group relative to the general population: *β* = 1.5 or 3.

**Table 1:** Weibull distribution parameters governing simulated population and study data under six simulation scenarios, defined by combinations of three Weibull shape specifications and two log (study/population) hazard ratios *β*.

Data	Parameter	1: Weibull	2: Decreasing cause‐specific relative hazard	3: Rapidly decreasing cause‐specific relative hazard
General population	*α* ^0^	(1.7, 1.7)	(1.5, 2)	(1.5, 4.5)
(*n*0 = 1000)	*λ* ^0^	(0.0015, 0.0022)	(0.0015, 0.0022)	(1.5 × 10^−3^, 1.5 × 10^−7^)
	Mean	24.04	17.58	26.96
Study group	*α*	(1.7, 1.7)	(1.5, 2)	(1.5, 4.5)
(*n* = 500)	*λ*	(0.0015*e* ^*β*^, 0.0022)	(0.0015*e* ^*β*^, 0.0022)	(0.003*e* ^*β*^, 0.001)
*β* = 1.5	Mean	14.32	14.22	19.78
Study group	*α*	(1.7, 1.7)	(1.5, 2)	(1.5, 4.5)
(*n* = 500)	*λ*	(0.0015*e* ^*β*^, 0.0022)	(0.0015*e* ^*β*^, 0.0022)	(0.003*e* ^*β*^, 0.001)
*β* = 3	Mean	6.72	8.09	9.20

The Weibull parameters and mean survival implied by these six combinations of scenarios are summarised in Table 1. Model 2 with *β* = 1.5 approximately reflects the mortality rates in our motivating application to the UK ICD cohort. For each scenario and simulation replicate, two datasets were generated, the first with 1000 observations corresponding to times of death for the general population and the second with times of death for 500 patients from the study cohort. Censoring times were generated from an exponential distribution, up to 10 years, at which time all surviving patients were censored. The exponential rate was set to 0.15 if *β* = 1.5, or 0.4 if *β* = 3, to give a similar pattern of censoring to the ICD cohort, where 15% of events were observed by 10 years.

**Figure 1:**
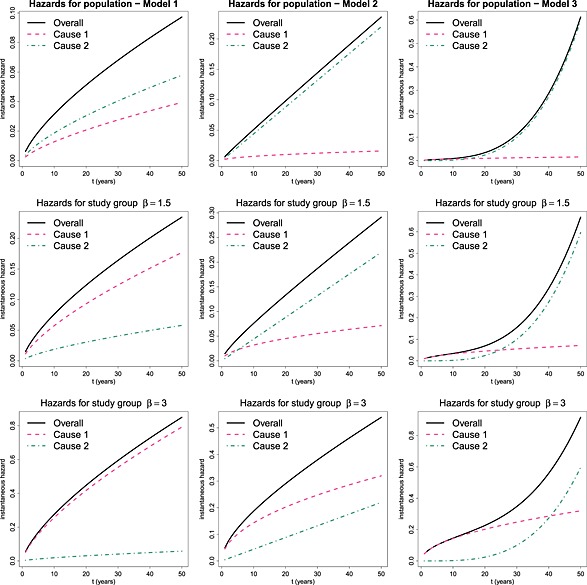
Hazard functions for data simulated for the population (first row) and study group with *β* = 1.5 (second row) and *β* = 3 (third row); under models 1 (left), 2 (middle) or 3 (right).

The hazard functions for the models described in the preceding text are presented in Figure 1. The population data are represented in the top row, while the middle and bottom rows represent the study data whose cause‐specific log hazard ratio *β* compared to the population is 1.5 and 3, respectively. Notice that in Model 1 (left column), the hazard related to other causes is larger than the hazard of interest in the general population, but that direction is reversed in the study cohort. In models 2 and 3 (middle and right columns), the hazard related to the cause of interest is very small in the population data, but increases in the study cohort at a rate that depends on*β*. A characteristic of Model 3, that is plausible for some situations, is that the hazard for the cause of interest in the study group is higher compared to other causes for a certain period at the beginning of the study, which represents the time immediately after presentation, but as the patient gets older, the hazard of dying from other causes becomes predominant.

For each of the six scenarios, we simulated 50 replicate datasets and fitted three different models:

1. Poly‐Weibull (4): this is the true model and assumes a cause specific effect, in that the hazard for mortality from the disease of interest differs between the study and general populations, but the hazards for other causes do not.
2. Weibull model fitted to the combined data, with joint likelihood defined by a product of population and study likelihoods with common shape *α* and scales *λ* and *e* ^*β*^ *λ*, respectively. Thus, the hazard for *overall* mortality is assumed proportional between the study and general population.
3. Semi‐parametric ‘Cox‐like’ model: using a similar joint likelihood with proportional hazards (for all causes) between the population and the study group. The baseline hazard is a piecewise‐constant function with change points at every observed death time. This model is described in more detail by Demiris and Sharples 6 and Jackson *et al*. 2. Like the standard Cox model, this assumes proportional hazards while making minimal assumptions about the baseline hazard.

### 3.2 Prior distributions

We used weakly informative priors corresponding to beliefs expressed on an easily interpretable scale. For all poly‐Weibull and Weibull models, we used Uniform(0,100) priors for the scale (1/*λ*_*k*_ or 1/*λ*). This expresses the belief that patients cannot survive more than about 60 years after entry to the study (simulated patients were assumed to be aged 60 at entry)—because even when *α* is at its prior limits, the implied mean cause‐specific survival time of (1/*λ*)Γ(1 + 1/*α*) cannot be more than about 60. We expect the hazard for both arrhythmia‐related death and other causes of death to increase through time. Therefore, we use weakly informative Normal priors for log(*α*_*k*_) (or log(*α*)) with a mean of 0.5 and standard deviation of 0.78 = (log(log(100)/ log(2) + 1) − 0.5)/Φ^−1^(0.975), which implies an expected hazard ratio of 1.5 for a doubled time since age 60 years, with 95% credible interval approximately between 0.64 and 100. For the log hazard ratio of mortality from the disease of interest between the general population and the patients of interest, we use Normal priors with mean 0 and standard deviation of 2.5, which gives a 95% credible interval for the hazard ratio of between 1/150 and 150. To aid the identifiability of the two components of the poly‐Weibull model, we constrained the scale parameters such that *λ*_1_<*λ*_2_, as suggested by Demiris *et al*. 8. For the semi‐parametric model, independent Gamma(*c* *μ*,*c*) priors were used for the baseline hazards, as in 6, with *c* = 200 to ensure a high variance, and the mean *μ* set to the true hazard.

The models were fitted using MCMC algorithms in WinBUGS 9, using the WBDev add‐on 10 to implement the poly‐Weibull distribution. An example of the WinBUGS code required for these models is given in Appendix A.

### 3.3 Results

Tables 2 and 3 show, for each of six scenarios, the mean (over simulation replicates) of the posterior mean expected survival, the corresponding mean absolute (and percentage) bias, and the coverage of the 95% credible intervals.

**Table 2:** Bias and coverage in point and interval estimates of expected survival when *β* = 1.5.

Model		True	Weibull	Cox‐like	Poly‐Weibull
1: Weibull	Mean	14.32	14.31	14.71	14.76
	Bias (%)		−0.01 (−0.1%)	0.39 (2.7%)	0.44 (3.1%)
	Coverage		0.94	0.98	0.96
2: Decreasing cause‐specific	Mean	14.22	13.40	13.75	14.16
relative hazard	Bias (%)		−0.82 (−5.8%)	−0.47 (−3.3%)	−0.06 (−0.4%)
	Coverage		0.78	0.96	0.72
3: Rapidly decreasing cause‐	Mean	19.78	14.19	17.10	19.15
specific relative hazard	Bias (%)		−5.62 (−28.4%)	−2.68 (−13.5%)	−0.63 (−3.20%)
	Coverage		0.00	0.80	0.90

**Table 3:** Bias and coverage in point and interval estimates of expected survival when *β* = 3.

Model		True	Weibull	Cox‐like	Poly‐Weibull
1:Weibull	Mean (95% CI)	6.72	6.76	7.11	6.95
	Bias (%)		0.04 (0.6%)	0.39 (5.8%)	0.23 (3.40%)
	Coverage		0.92	0.96	0.84
2: Decreasing cause‐specific	Mean (95% CI)	8.09	7.08	7.78	7.68
relative hazard	Bias (%)		−1.01 (−12.5%)	−0.31 (−3.8%)	−0.41 (−5.1%)
	Coverage		0.52	0.96	0.82
3: Rapidly decreasing cause‐	Mean (95% CI)	9.20	6.77	9.13	8.71
specific relative hazard	Bias (%)		−2.43 (−26.4%)	−0.07 (−0.8%)	−0.49 (−5.3%)
	Coverage		0.00	1.00	0.86

**Figure 2:**
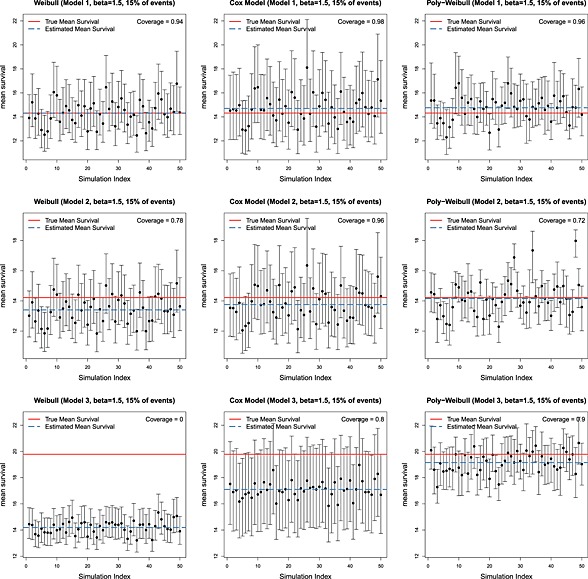
Posterior means and 95% credible intervals for expected survival under 50 simulation replicates, for data simulated under three different models and *β* = 1.5. True value shown is in red, and mean of 50 posterior means is shown in blue.

**Figure 3:**
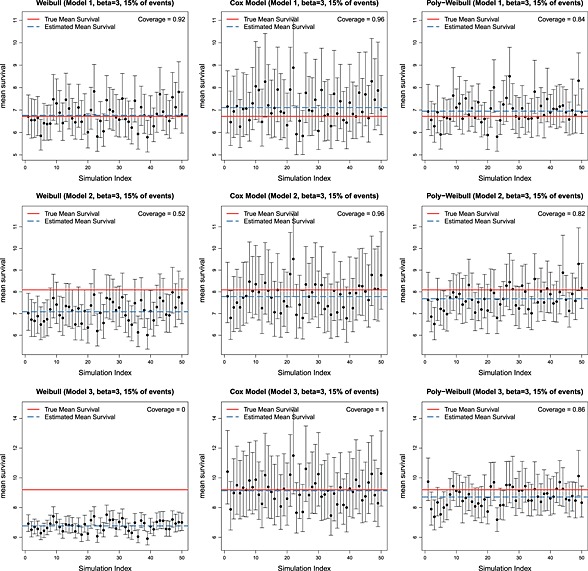
Posterior means and 95% credible intervals for expected survival under 50 simulation replicates, for data simulated under three different models and *β* = 3. True value shown is in red, and mean of 50 posterior means is shown in blue.

Independently of the value of *β*, when the data are simulated from the Weibull model 1, there is negligible bias and coverage close to the nominal 95% for all fitted models. This is expected, because the true model has the same shape parameter for both causes of death and thus reduces to a Weibull model for overall mortality. When the data arise from Models 2 and 3, as the difference between the cause‐specific shape parameters increases, the bias resulting from fitting the Weibull model also increases. This is illustrated in Figures 2 and 3, which show the posterior mean and 95% credible interval for expected survival for each simulation replicate. The red solid horizontal line represents the true value, and the blue dashed line is the mean (over 50 replicates) of the posterior means. The poly‐Weibull model gives the least biased estimates for *β* = 1.5. For *β* = 3, the Cox‐like model also has reasonably low biases— but although the posterior means of expected survival are not far from the true value, the uncertainty around the estimates is always bigger compared to the other models, due to the need to estimate a different baseline hazard for every interval between event times (Figures 2 and 3). This can lead to interval estimates with excess coverage (e.g., Table 3, Model 3), although there is some uncertainty in these estimates of coverage, due to only performing 50 simulation replicates. Most noticeable is the bias introduced when ignoring the cause specific effect by assuming a Weibull model. When the contribution of the cause of interest to overall mortality is rapidly decreasing through time (Model 3), and *β* = 1.5, the Weibull model underestimates the mean survival by an average of 5.6 years. Likewise if *β* = 3, mean survival is underestimated by almost 3 years. In both cases, this is a bias of the order of 30%, which would often make a substantial difference to policy decisions. In contrast, the biases in the estimates obtained using the Cox‐like model are much lower, with mean survival underestimated by at most 2.68 years. Thus, the flexibility of the baseline hazards in the Cox model will, to some extent, compensate for the misspecified assumption of proportional hazards for overall survival to give minimally biased estimates of overall survival. The price for this is a reduction in efficiency and loss in precision (demonstrated by coverage probability of 1), which may be important if the data set is small.

**Figure 4:**
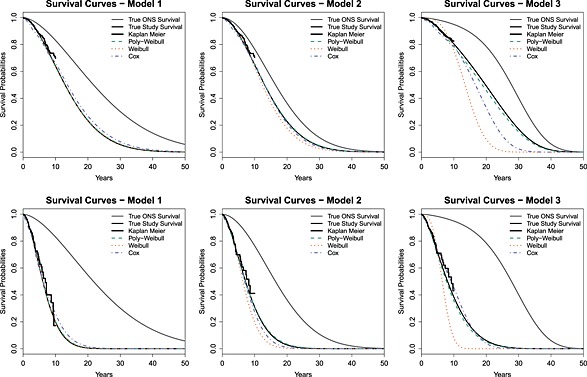
True and fitted survival curves for one simulation replicate, under true models 1, 2, and 3, and *β* = 1.5 (top) or *β* = 3 (bottom)

These results are also illustrated in Figure 4, which compares the true survival curves to a fitted survival curve for a representative simulation replicate. There is a good fit by all models when the data are generated from Model 1, whereas the mean survival is underestimated by the Weibull model when the data are generated from Model 3. The underestimation is particularly evident when survival is extrapolated. The pattern for bias in the Cox‐like model is less clear in our simulations, reflecting the higher variance in these estimates.

Our simulation study has focused on the specific case of the use of Weibull and Cox models for overall survival when the contribution of the cause‐specific risk of interest is decreasing over time, so that overall survival is correctly modelled by a poly‐Weibull distribution. While this study gives an indication of the patterns of bias and efficiency when estimating mean survival, it does not comprehensively investigate the degree of bias and efficiency. The study demonstrated that for model 3, in which a doubling of time relates to a doubling of risk for the rapidly increasing hazard, the frequently used Weibull distribution can result in bias of the order of 30*%*, while the Cox model will be more accurate (at most 13.5*%* bias in our simulations). We note that the extent of bias due to changing risk contribution is primarily influenced by the shape parameters *α*. Detailed examination of these parameters (or analogous parameters in alternative survival functions) is most likely to provide insight into the implications of ignoring cause‐specific hazards. Additionally, the simulation study does not investigate a wide range of possible survival distributions. For example, the poly‐Weibull may give poor estimates of overall survival if the data were generated according to, say, a poly‐Gompertz distribution. Therefore, assessment of model fit to both short‐term and long‐term data sources is important, although not all assumptions about extrapolated parameters can be checked that way, and a more comprehensive simulation study could be worthwhile.

## 4 Application to implantable cardioverter defibrillators

### 4.1 Implantable cardioverter defibrillators cohort and population data

The simulation study showed the potential for bias when study group survival is extrapolated by applying overall instead of cause‐specific hazard ratios to population data when the cause of interest is not a constant proportion of all cause mortality through time. Here, we apply the same models to a study of ICD in a cohort of patients with cardiac arrhythmia who have survived a ‘sudden cardiac death’ event. Thus, arrhythmia‐related deaths are cause *k* = 1.

**Figure 5:**
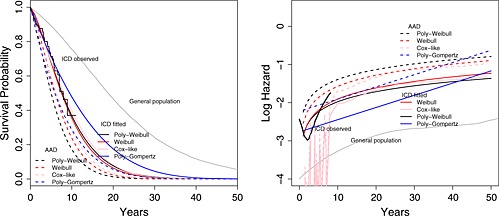
Survival curves (left) and log hazards (right) for implantable cardioverter defibrillators (ICD) patients (short‐term observed and long‐term fitted), anti‐arrhythmic drugs (AAD) patients (long‐term fitted) and general population (observed) using the Weibull, Cox‐like, poly‐Weibull and poly‐Gompertz models.

The study data consist of 535 patients implanted with ICDs between 1991 and 2002 (244 and 291 implants from Papworth and Liverpool hospitals, respectively). There were 81 observed deaths, and the follow‐up was at most 10 years post implant, with 75% of the sample having follow‐up below 3.5 years. The empirical survival curve is illustrated in Figure 5 (left). Causes of death were not recorded. The average age at implant was 60 years, and men represented 81% of this cohort. The empirical hazard rate fluctuated around an approximately constant value during the observed follow‐up period (Figure 5, right); however, it is unlikely to remain constant in the long term because we expect mortality rates to increase with age. As in Demiris and Sharples 6, we assume that the distributional family governing the survival of ICD patients is the same as that of the general population in the UK matched by age and sex. While Demiris and Sharples 6 assumed proportional hazards for all causes of death between the ICD cohort and the general age–sex matched population, we assume that the ICD patients have (higher) proportional hazards for arrhythmic deaths, but identical mortality for other causes of death, compared to the population.

**Figure 6:**
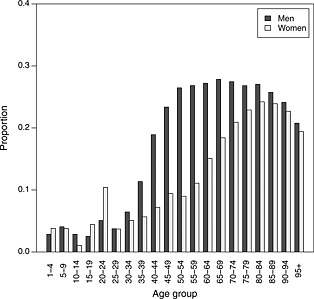
Proportion of deaths in the UK population in 2002, which were due to cardiac arrhythmia

The UK population survival data were generated using annual life tables, by age and sex, published by the UK Office for National Statistics (ONS) (http://www.ons.gov.uk/ons/taxonomy/index.html?nscl=Interim+Life+Tablesdownloaded16thDec2012). We also obtain population data on causes of death from the UK ONS, in particular their table of deaths by underlying cause, for diseases of the circulatory system. In an informal discussion with a clinical expert (Andrew Grace, personal communication), we identified which coded causes of death in this table were thought to be arrhythmia related, thus potentially preventable by ICD implantation. Hence, we computed the proportion of deaths, which were related to arrhythmia, illustrated in Figure 6 for sex and 5‐year age groups. For each ICD patient, we generated survival times from 20 controls from the general population, matched by age and sex. The survival time for each control was generated by randomly sampling their annual survival status, year by year, using the survival probabilities from the published life tables. A cause of death (classified as arrhythmia or non‐arrhythmia) was similarly generated for each control by random sampling using the data in Figure 6. Twenty controls were judged to be sufficiently many that we can assume negligible sampling uncertainty in the population data relative to the study data.

Note that over the age range of interest (≥ 60 years), the proportion of deaths (potentially) related to arrhythmia is fairly constant for men but is increasing for women (Figure 6). This suggests that assuming proportional all‐cause hazards may give greater biases for women than men in extrapolated survival estimates.

### 4.2 Models for combining cohort and population data

In previous sections, we extrapolated the survival of a single group of patients at high risk of death from a specific cause, using population survival patterns. We do this here for the ICD cohort using the previously described models: a Weibull with proportional all‐cause hazards between the ICD cohort and the population data, a Bayesian semi‐parametric Cox‐like model, and a poly‐Weibull model with proportional hazards for arrhythmia mortality. The same prior distributions were used as in the simulation study (Section 3.2). As a test of the Weibull assumption for cause‐specific hazards, we fitted an alternative polyhazard model based on the Gompertz distribution. This ‘poly‐Gompertz’ model has the same definition as in Equation (2), but with cause *k* hazard *λ*_*k*_ exp(*α*_*k*_ *t*) instead of $\lambda_{k}\alpha_{k}t^{\alpha_{k}-1}$, so that we again assume proportional cause‐specific hazards, but with a differently shaped hazard trajectory. Similar principles were used to derive its priors.

### 4.3 Comparisons between competing treatments

In cost‐effectiveness analysis, we typically need to compare expected survival between patients receiving different interventions. Thus, we add a third data source to our ICD patients and our general population. In this example, we compare patients implanted with ICDs to patients treated with AAD. A summary treatment effect is available from a meta‐analysis of three randomised controlled trials 5 comparing ICDs with AAD for the secondary prevention of sudden cardiac death. For the outcome of death from any cause, the hazard ratio (ICD : AAD) was 0.72 (95% CI: 0.60–0.87). For death from arrhythmia‐related causes, the published hazard ratio was 0.50 (95% CI: 0.37– 0.67). We can incorporate this information straightforwardly in our previous models as a prior distribution, assuming only the hazard related to arrhythmia deaths is affected by the treatment. Thus, using the notation of Section 2, the hazards for the population, ICD and AAD groups under the polyhazard models are, respectively, given by

(5) $$\begin{array}{cc} h_{p}(t|\alpha ,\lambda ) & =h_{p}^{1}(t|\alpha_{1},\lambda_{1})+h_{p}^{2}(t|\alpha_{2},\lambda_{2})\\ h_{s}(t|\alpha ,\lambda ,\beta ) & =e^{\beta }h_{p}^{1}(t|\alpha_{1},\lambda_{1})+h_{p}^{2}(t|\alpha_{2},\lambda_{2})\\ h_{\text{AAD}}(t|\alpha ,\lambda ,\beta ,\gamma ) & =e^{\gamma +\beta }h_{p}^{1}(t|\alpha_{1},\lambda_{1})+h_{p}^{2}(t|\alpha_{2},\lambda_{2}), \end{array}$$

where *γ* is the log hazard ratio (AAD : ICD) related to *arrhythmia‐related deaths*. Using the meta‐analysis summary statistic given in the preceding text, we set the prior for this parameter to be *N*(0.693,0.148^2^).

For the Weibull and Cox‐like models, which assume proportional overall hazards, we have population hazard *h*_p_(*t*|*λ*,*α*), and

$$\begin{array}{cc} h_{s}(t|\lambda ,\alpha ,\beta ) & =e^{\beta }h_{p}(t|\lambda ,\alpha )\\ h_{\text{AAD}}(t|\lambda ,\alpha ,\beta ,\gamma ) & =e^{\beta +\gamma }h_{p}(t|\lambda ,\alpha ), \end{array}$$

where *γ* here is the log hazard ratio (AAD : ICD) related to *all causes of death*. For these models, the prior for *γ* was *N*(0.3285,0.093^2^), also obtained from the meta‐analysis summary hazard ratio.

The outcome of interest is the life years gained (LYG) from a policy of ICD implantation compared to AAD treatment, which is the difference between the expected survival for patients under each treatment. These are presented in Table 5, and the estimates for the basic parameters are given in Table 5 of the online Appendix B.

### 4.4 Comparison of model fit

The poly‐Weibull distribution fits the ICD study data reasonably well overall (Figure 5), and by gender (Supplementary Figure 7). Supplementary Figure 8 indicates that the Weibull distribution gives an adequate fit to the general population survival data by cause and gender. This suggests the poly‐Weibull‐based model is reasonable for use in extrapolation. The estimates of the survival of ICD patients from the other three models agree with the Kaplan–Meier estimate well. Although note that short‐term fit is only a limited guide to long‐term model adequacy—the poly‐Gompertz models show substantially poorer fit to the *long‐term* general population data for both arrhythmic and non‐arrhythmic death times separately and both genders (Figure 8). Figure 5 also suggests the hazard over 40 years is underestimated (or survival overestimated) by the poly‐Gompertz model due to the influence of the data from the first 5 years on the shape of the trajectory.

The polyhazard models are of identical complexity, so improvements in fit can be quantified by reductions in the posterior mean deviance $\bar{D}$ corresponding to likelihood contributions (1) (for each cause of death separately) and (3). While $\bar{D}$ for the study data is 16 lower for the poly‐Gompertz model compared to the poly‐Weibull, the corresponding $\bar{D}$ for long‐term general population arrhythmic and non‐arrhythmic deaths is 270 and 98 higher, respectively, indicating a poorer overall fit for the poly‐Gompertz. This suggests the poly‐Gompertz would give less plausible extrapolations of long‐term survival of our study patients, despite the marginally better short‐term fit.

### 4.5 Comparison of expected survival

The estimated LYG is greater by more than 1 year under the poly‐Weibull model, compared to the two models that ignore causes of death (Table 4). This difference between the models is most apparent in the estimated survival for the AAD patients, which is also influenced by the meta‐analysis through the parameter *γ*. These results are also demonstrated in Figure 5, which shows the fitted survival curves and log hazards for both the ICD and AAD patients. The poly‐Gompertz gives much higher estimates of expected survival and LYG, and its poorer fit to the long‐term data suggests that these are implausible. Under the Cox‐like model, the expected survival for both the treatment groups is greater compared to the Weibull, but the incremental survival is not materially affected—this suggests that modelling the baseline survival more flexibly does not completely alleviate bias due to ignoring cause‐specific mortality.

**Table 4:** Posterior mean (posterior standard deviation) of expected survival for patients receiving implantable cardioverter defibrillators (ICDs) and anti‐arrhythmic drugs (AAD), by sex.

Parameter	Survival models			
	Weibull	Cox‐like	Poly‐Weibull	Poly‐Gompertz
Overall
ICD mean survival	8.88 (0.71)	9.41 (0.78)	9.18 (0.75)	11.54 (1.03)
AAD mean survival	7.06 (0.73)	7.43 (0.81)	6.06 (0.80)	7.62 (1.15)
Life years gained	1.82 (0.49)	1.98 (0.53)	3.12 (0.61)	3.92 (0.78)
Male
ICD mean survival	8.52 (0.73)	9.04 (0.81)	8.72 (0.75)	11.00 (1.03)
AAD mean survival	6.79 (0.73)	7.13 (0.82)	5.80 (0.78)	7.31 (1.14)
Life years gained	1.73 (0.47)	1.91 (0.52)	2.91 (0.58)	3.69 (0.74)
Female
ICD mean survival	10.11 (1.95)	9.87 (2.14)	9.96 (1.97)	14.54 (3.25)
AAD mean survival	8.22 (1.66)	7.92 (1.81)	6.85 (1.61)	9.87 (3.14)
Life years gained	1.89 (0.62)	1.95 (0.65)	3.11 (0.76)	4.67 (1.05)

Recall that the priors were weakly informative and based on beliefs expressed on a natural scale (Section 3.2). The results were found to be robust to alternative standard choices, except in the case of the polyhazard models for women, where the mean survival was sensitive to the assumed prior variance, because these models are weakly identifiable given there are only 104 women in the ICD data, 12 of whom died. For example, in the poly‐Weibull, the mean ICD survival for women was 11.13 under a N(0, 1000) prior for the log(*λ*_*k*_), which is effectively uniform on a different scale although permits unnaturally high survival times.

As previously noted, Figure 6 shows that the proportion of deaths by age for men that are due to arrhythmia is approximately constant from age 60 years, but increases with age for women. Because of this, the effect of treatment might differ by gender (see application in Section 4). The expected survival and LYG for each gender individually are presented in Table 4. Again, the LYG are greater for both men and women if assuming a cause specific rather than an overall mortality differential between the general and study populations. For women, this difference is approximately 65% higher, which implies that in a cost‐effectiveness analysis, the cost per year of life gained would be almost 60% of that estimated by the models that ignore causes of death. However, even for men, there is a difference of about a year between the estimated survival of AAD patients after accounting for cause‐specific mortality. Interpretation of the results in this particular example is not straightforward. The overall mean survival for both men and women is similar for the Weibull, Cox‐like and poly‐Weibull models, which was expected for men but not for women. Failure to identify the increasing proportion of deaths attributable to arrhythmias in women may be due to the small sample in the ICD cohort (12 deaths in 104 women), which may have caused identification problems in the poly‐Weibull model. Sensitivity to prior distributions highlighted in the preceding text is also an indication of the lack of information in this data set. In this example, the increase in LYG for both men and women in the cause‐specific analysis results from the difference in estimated mean survival for the AAD patients, which is driven by both the published arrhythmia‐specific hazard ratio and the proportion of all cause mortality that can be influenced by an ICD. Reliable analysis will depend on correct classification of the causes of death that can be influenced by the ICD and an estimate of the arrhythmia‐specific hazard ratio *γ* that is consistent with these causes. The large difference between the Poly‐Weibull and Weibull observed in this example may be due to inconsistency in the definition of ‘arrhythmic mortality’ between the cause‐specific hazard ratios published by Connolly *et al*. 5 and our clinical colleague's informal identification of arrhythmia‐related causes of death in the general population—thus, the model may not be adjusting for the difference between the population and study patients properly.

### 4.6 Sensitivity to classification of causes of death

To investigate this potential misclassification, we performed two sensitivity analyses, in which we randomly reclassified 10% and 20% of the arrhythmia‐coded deaths in the general population as non‐arrhythmic, and fitted the poly‐Weibull model again. This resulted in reductions in the expected survival of both treatment groups, and a 4% and 8% reduction in the expected LYG from ICD, respectively, (Table 6 of the online Appendix B). This is as expected, because with fewer deaths in the long term, which might have been affected by the treatment, the effect of treatment on extrapolated survival will be lower. The same reductions in expected LYG are observed for the same analyses by gender. However, the estimated survival of male AAD patients is still about a year lower after accounting for cause‐specific mortality using the poly‐Weibull.

Another possible explanation is that the ICD study cohort may be at greater risk than the general population from other causes of death in addition to arrhythmia. Labelling these causes ‘cause 3’ (and omitting the parameters *α*,*λ* on the right‐hand side for clarity), the population survival in Equation (5) would become

$$h_{p}(t|\alpha ,\lambda )=h_{p}^{1}(t)+h_{p}^{3}(t)+h_{p}^{2}(t).$$

The parameter *β* would then represent the log hazard ratio between the population and cohort for these causes combined with arrhythmia, so that

$$h_{s}(t|\alpha ,\lambda ,\beta )=e^{\beta }\{h_{p}^{1}(t)+h_{p}^{3}(t)\}+h_{p}^{2}(t).$$

However, assuming the published effect *γ* of treatment (AAD or ICD) only applies to cause 1 mortality, the true hazard for the AAD group would then be

$$h_{\text{AAD}}(t|\alpha ,\lambda ,\beta ,\gamma )=e^{\gamma +\beta }h_{p}^{1}(t)+e^{\beta }h_{p}^{3}(t)+h_{p}^{2}(t).$$

In order for our previous model (5) to apply in this situation, with $h_{p}^{1}(t)$ replaced by $h_{p}^{1}(t)+h_{p}^{3}(t)$, *γ* would need to represent the effect of treatment on causes 1 and 3 combined, which would be weaker than the published arrhythmia‐specific effect. Because the AAD group has poorer survival, this would explain the possible underestimation of the AAD‐specific survival in the poly‐Weibull model.

## 5 Discussion

Extrapolations of survival of patients from randomised trials or study cohorts can often be facilitated with official survival statistics from the general population. This requires carefully characterising the differences between the population and the study patients. We have examined the circumstances under which simply assuming proportional hazards for all‐cause mortality can lead to bias when estimating expected lifetime survival. If the study patients are at a higher risk of death from some identifiable cause, and the contribution of that cause to overall mortality is not constant through time, then the hazards for overall mortality will not be proportional. The resulting bias can be alleviated by using proportional hazards for the cause of interest, assuming that cause‐specific survival times are available in *either* the population or the study data. If causes of death are unpublished in just one of the two datasets, as in our example, then overall survival in *that* dataset can be modelled with a polyhazard distribution, whose hazard is defined as the sum of cause‐specific hazards.

In our application to ICD, ignoring the cause‐specific hazard ratio resulted in a lower estimate of the gain in mean survival for women, for whom the proportion of deaths due to arrhythmia increases with age. The corresponding LYG from ICD implantation as compared to AAD was approximately 65% higher after accounting for cause‐specific survival. Hence, the cost per life year gained, required for policy‐making, would be substantially higher under the Weibull model, which is likely to be misspecified. Care should be taken in the interpretation of these estimates, because the size and direction of bias will be influenced both by the varying proportion of arrhythmia deaths and by other model assumptions, such as the fit of the Weibull model, the accuracy of the classification of causes of death, and the assumption that the causes that distinguish the ‘arrhythmia’ cohort from the population are the same causes affected by the published ‘arrhythmia‐specific’ treatment effect. In the ICD example, both men and women ICD patients had lower estimated gain in survival time compared to AAD patients using the Weibull model (Table 4), despite the largely constant proportion of deaths classified as arrhythmia related (Figure 6). It is possible that assumptions regarding the classification of causes of death and the partitioning of the overall hazard into cause‐specific hazards influenced these estimates of survival, as discussed in the previous section, particularly because they are extrapolated. It is difficult to distinguish the effect of each questionable assumption in extrapolation. This emphasises the need for sensitivity analyses, and careful and consistent categorisation of causes of death in survival data. In addition, a more comprehensive simulation study, including a wider range of survival distributions and patterns of risk contributions, would clarify the extent of potential bias and efficiency resulting from ignoring cause‐specific hazards.

The Weibull distribution is particularly convenient for modelling cause‐specific survival because its proportional hazards property gives an intuitive scale for expressing the differences between populations, and treatment effects are typically published as hazard ratios. However, the Weibull family has a limited range of hazard trajectories, which may not express, for example, the rapidly increasing hazards seen in older populations. Different distributions can be used to generate polyhazard models. The Gompertz was investigated in our application, although found to be less suitable for our data. A semi‐parametric Cox‐type model with competing hazards would allow a more flexible baseline hazard for describing general population survival patterns, although any assumptions about proportionality of hazards should still be checked. Our simulation also suggested that bias can be alleviated by using a semi‐parametric model, while still assuming proportional overall hazards, but at the cost of reduced precision compared to using the true parametric survival distribution. In addition, our poly‐Weibull formulation assumes that hazards for different causes are additive and independent of each other, an assumption that is again impossible to test and may require sensitivity analysis.

As in any Bayesian application, priors may be influential for smaller sample sizes and models with more parameters. If substantive prior knowledge exists, as it does for human survival, it should be included. In our example, expected survival for women appeared to be overestimated by a year under an unrestricted diffuse prior, which placed about 50% prior mass on mean survival times of more than 1000 years.

If the interest is in comparing expected survival between different interventions, then the intervention effect will typically be only available from randomised trials with short‐term follow‐up. Comparing survival over longer periods requires untestable assumptions, such as the treatment effect remaining constant after the end of the trial, or instantly reducing to zero, or decreasing gradually through time. Again, these can only be explored through sensitivity analysis. Note also that the example we present involves a joint analysis of randomised data, included as a prior on an intervention effect *γ*, and observational data. The posterior distribution of the intervention effect is however identical to the prior, because the observational data do not contribute any likelihood. *γ* can therefore be interpreted as a causal effect of the randomised intervention. In other examples, we might want to estimate *γ* from a combination of randomised and observational data. Then for a causal interpretation, we must ensure, for example, that all confounders have been controlled for.

Finally, it is important to bear in mind the context in which these issues arise. Survival patterns in different subgroups are almost invariably a feature of health economic decision models, but other primary and secondary data sources are often included. In particular, disease progression and its relationship with survival is typically modelled, along with costs and utility assessments associated with different health states. The relationship between cause‐specific survival methods and other components of the decision model should be the subject of future work.

## Supporting information

Supporting info item

## Appendix A WinBUGS model code

### A.1

Here, we present the code for both the two‐component poly‐Weibull model, discussed in Section 2, and a Weibull model, which ignores the cause specific effect.
