# Supplementary material for: Survival extrapolation in the presence of cause specific hazards
Source: Stat Med. 2015 Mar 5;34(5):796–811. doi: 10.1002/sim.6375 (PMC4847642; doi:10.1002/sim.6375)
Supplement: Supplementary file 1 — Supporting info item [file SIM-34-796-s001.pdf]

B. Supplementary Tables and Figures - Application

This appendix presents basic parameter estimates for each model for everybody and by sex (Table 5), followed by the results of the sensitivity analysis to misclassification of causes of death (Table 6), empirical and fitted survival curves and hazard functions for men and women, respectively (Figure 7), and goodness of fit of polyhazard models to the long-term general population data (Figure 8).

Table 5. Posterior means and 95% credible intervals for parameters in each model, for everybody and by sex

Model	Parameter	Overall	Male	Female
**Weibull**	α	1.4209 (1.3990, 1.4430)	1.4319 (1.4080, 1.4560)	1.5732 (1.5190, 1.6290)
	β	1.2587 (1.0290, 1.4740)	1.2181 (0.9672, 1.4550)	1.6639 (1.0370, 2.2210)
	λ	0.0112 (0.0104, 0.0121)	0.0122 (0.0112, 0.0132)	0.0044 (0.0036, 0.0054)
**Cox-like**	β	1.1996 (1.1222, 1.2784)	1.1488 (1.0658, 1.2343)	1.6412 (1.4412, 1.8569)
**Poly-Weibull**	α_1	1.3692 (1.3290, 1.4110)	1.3945 (1.3520, 1.4380)	1.4834 (1.3970, 1.5730)
	α_2	1.4412 (1.4160, 1.4660)	1.4453 (1.4190, 1.4720)	1.6066 (1.5350, 1.6720)
	β	1.2742 (1.0520, 1.4800)	1.2575 (1.0320, 1.4880)	1.6578 (1.0560, 2.1590)
	λ_1	0.0034 (0.0029, 0.0040)	0.0036 (0.0031, 0.0042)	0.0014 (0.0010, 0.0019)
	λ_2	0.0078 (0.0071, 0.0085)	0.0086 (0.0078, 0.0094)	0.0031 (0.0024, 0.0040)
**Poly-Gompertz**	α_1	0.0319 (0.0316, 0.0322)	0.0328 (0.0324, 0.0332)	0.0377 (0.0371, 0.0383)
	α_2	0.0320 (0.0317, 0.0323)	0.0331 (0.0327, 0.0335)	0.0380 (0.0374, 0.0386)
	β	1.8108 (1.7025, 1.9267)	1.7413 (1.6270, 1.8665)	2.1230 (1.8823, 2.4350)
	λ_1	0.0071 (0.0070, 0.0072)	0.0079 (0.0077, 0.0080)	0.0038 (0.0037, 0.0040)
	λ_2	0.0187 (0.0185, 0.0189)	0.0209 (0.0206, 0.0211)	0.0106 (0.0103, 0.0108)

Table 6. Sensitivity of estimates of expected survival to different rates of misclassification of causes of death in the population data. Posterior means and 95% credible intervals from the poly-Weibull model.

	Proportion of deaths coded as arrhythmia which are wrongly classified		
	None	10%	20%
OVERALL			
ICD mean survival	9.18 (7.82, 10.78)	8.99 (7.67, 10.57)	8.83 (7.61, 10.29)
AAD mean survival	6.06 (4.64, 7.76)	5.99 (4.60, 7.68)	5.94 (4.60, 7.54)
Life years gained	3.12 (1.91, 4.31)	3.00 (1.83, 4.14)	2.89 (1.76, 3.98)
MALE			
ICD mean survival	8.72 (7.36, 10.29)	8.66 (7.33, 10.24)	8.60 (7.32, 10.13)
AAD mean survival	5.80 (4.42, 7.47)	5.80 (4.41, 7.46)	5.78 (4.42, 7.42)
Life years gained	2.91 (1.76, 4.03)	2.86 (1.74, 3.97)	2.82 (1.72, 3.90)
FEMALE			
ICD mean survival	9.96 (7.05, 14.68)	9.97 (6.94, 14.53)	9.87 (6.97, 14.20)
AAD mean survival	6.85 (4.51, 10.74)	6.89 (4.50, 10.61)	6.85 (4.53, 10.40)
Life years gained	3.11 (1.76, 4.75)	3.08 (1.76, 4.66)	3.02 (1.73, 4.59)

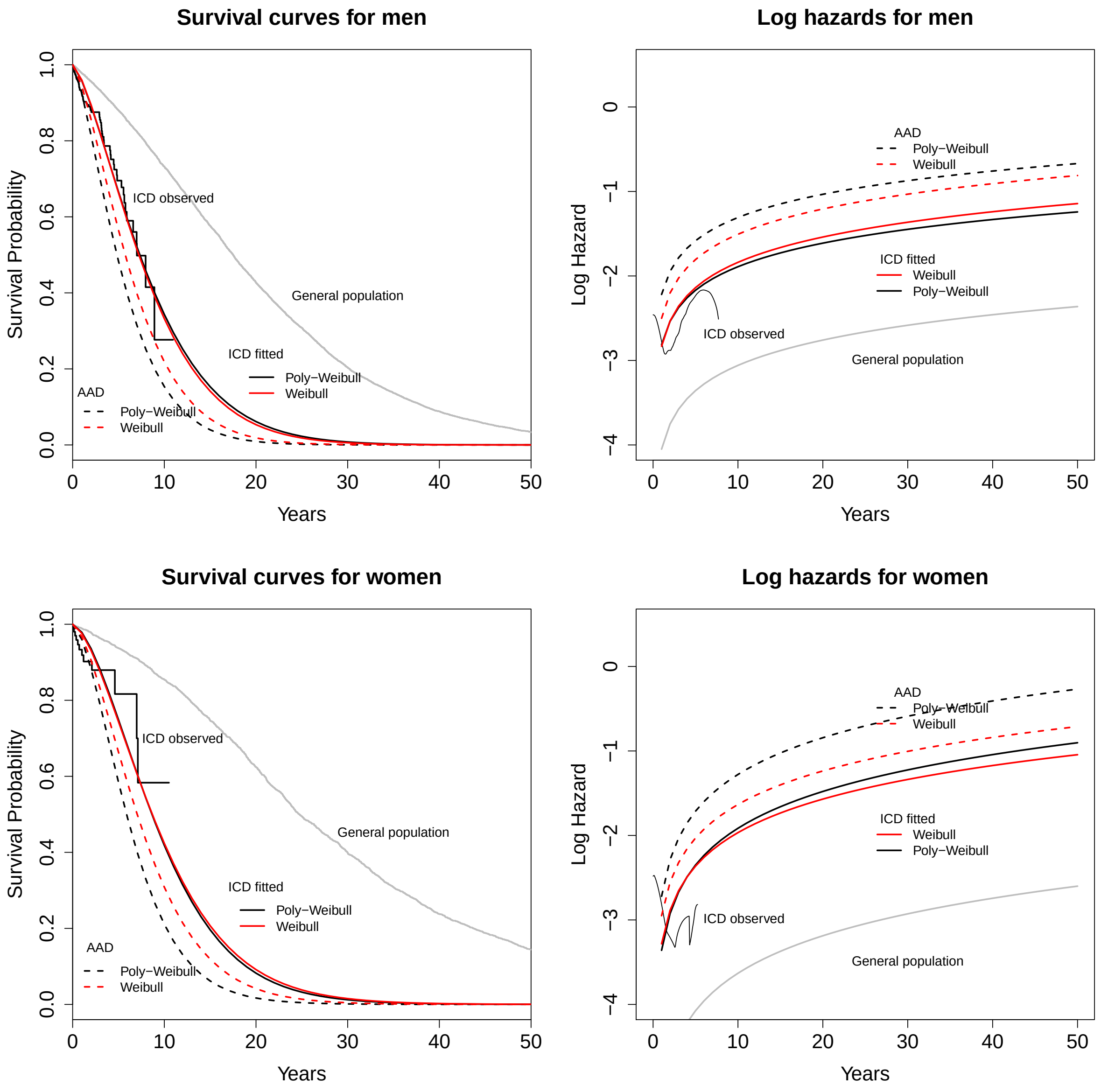

Figure 7. Survival curves and log hazards, for the ICD and AAD groups, fitted with the Poly-Weibull and Weibull models, for men (top) and women (bottom).

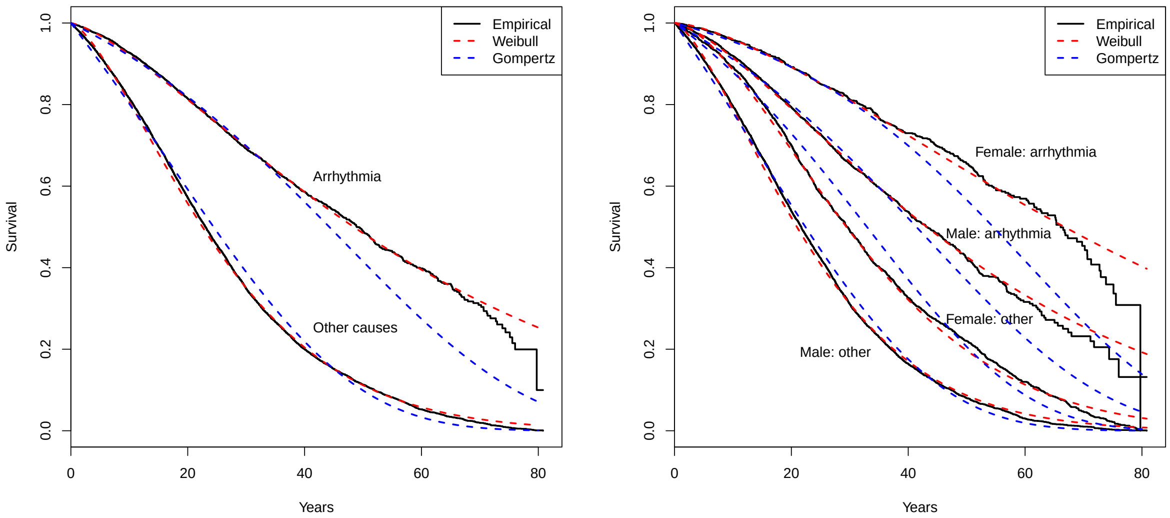

Figure 8. Fit of the Weibull and Gompertz distributions to the generated population cause-specific survival data (Kaplan-Meier estimates). Fitted values are the posterior means under the corresponding polyhazard models for the combined data. Left: overall, right: by gender.
